# Computer Mediated Social Comparative Feedback Does Not Affect Metacognitive Regulation of Memory Reports

**DOI:** 10.3389/fpsyg.2017.01433

**Published:** 2017-08-25

**Authors:** Joanne Rechdan, James D. Sauer, Lorraine Hope, Melanie Sauerland, James Ost, Harald Merckelbach

**Affiliations:** ^1^Department of Psychology, University of Portsmouth Portsmouth, United Kingdom; ^2^Department of Clinical Psychological Science, Faculty of Psychology and Neuroscience, Maastricht University Maastricht, Netherlands; ^3^Division of Psychology, University of Tasmania, Hobart TAS, Australia

**Keywords:** memory, meta-memory, social comparison, eyewitness memory, feedback

## Abstract

In two experiments, we investigated how social comparative feedback affects the metacognitive regulation of eyewitness memory reports. In Experiment 1, 87 participants received negative, positive, or no feedback about a co-witness’s performance on a task querying recall of a crime video. Participants then completed the task individually. There were no significant differences between negative and positive feedback groups on any measure. However, participants in both of these conditions volunteered more fine-grain details than participants in the control condition. In Experiment 2, 90 participants answered questions about a crime video. Participants in the experimental groups received either positive or negative feedback, which compared their performance to that of others. Participants then completed a subsequent recall task, for which they were told their performance would not be scored. Feedback did not significantly affect participants’ confidence, accuracy, or the level of detail they reported in comparison to a no feedback control group. These findings advance our understanding of the boundary conditions for social feedback effects on meta-memory.

## Introduction

Our memory for experienced events is the result of a reconstructive process that can be influenced by social factors (Bartlett, 1932). The presence of others can affect peoples’ confidence in their memory, and discussion of an event between two or more individuals can cause their accounts to converge (Wright et al., 2000, 2009; Shaw et al., 2007; Gabbert et al., 2012). These findings accord with the theory of social comparison, which posits that in the absence of objective means for assessing our opinions and abilities, we do so by comparing them to those of others (Festinger, 1954). When recalling events for which the ground truth cannot be determined, we may compare our memory to that of others who have experienced the same event in an attempt to produce an accurate account (Bless et al., 2001). Accuracy and informativeness of recall is monitored by metacognitive assessments, through which rememberers determine which details to report or withhold (Ackerman and Goldsmith, 2008). In two experiments, we examined how receiving social comparative feedback (i.e., information about others’ memory reports—or how our memory compares to that of others) affects the metacognitive processes that underlie memory reporting.

Memory reporting is governed by metacognitive processes, which allow individuals to monitor and control the information they volunteer (Koriat and Goldsmith, 1996). According to Ackerman and Goldsmith’s (2008) revised dual-criterion model, accuracy and informativeness are independent metacognitive criteria that individuals seek to satisfy when choosing which details to include in a memory report. Cutoffs for confidence in the accuracy of responses and their informativeness are personally established, and as such, vary from person to person. The precision of a response is adjusted/decreased (fine-to coarse-grain) until it meets these criteria, otherwise it is withheld. Coarse-grain information is less precise, and therefore more likely to be accurate than fine-grain information. For example, reporting that a perpetrator’s shirt is navy blue means that if the shirt were in fact forest green, the reported detail is erroneous. However, reporting that a perpetrator’s shirt is ‘dark’ encompasses a range of colors—the response is less detailed (no one color is specified) but less likely to be inaccurate.

In a series of studies, the authors gave participants varying degrees of control over the grain size of their responses to general knowledge questions. Results showed that when possible, participants adjusted the grain size of their responses to satisfy both accuracy and informativeness criteria. However, when participants were unable to satisfy both criteria, they were likely to violate the accuracy criterion to offer an informative, but less reliable answer. Given the option to withhold responses, participants did so in situations where desired levels of accuracy and informativeness could not be achieved through regulation of grain size. The experimental paradigm used by Ackerman and Goldsmith (2008; and previously by Goldsmith et al., 2002, 2005) offers an excellent means for examining metacognitive monitoring and decision-making regarding memory reporting. The theoretical model examines the role of confidence in the regulatory process, whereas the applied eyewitness literature has tended to focus on the role of confidence in diagnosing the accuracy of responses (Roberts and Higham, 2002; Vredeveldt and Sauer, 2015; Sauer and Hope, 2016).

Weber and Brewer (2008) applied Goldsmith et al.’s (2002) version of the model to examine the role of confidence in the strategic regulation of eyewitness memory. In two studies, they found that the level of detail provided by participants was strongly, positively correlated with their confidence in the accuracy of their fine-grain responses. Additionally, when participants were allowed to choose whether to report or withhold responses, they withheld information that failed to meet an implicitly established confidence criterion. In line with findings from previous research, these results indicate that confidence in the accuracy of fine-grain details recalled is a primary determinant of what participants choose to report (Goldsmith et al., 2002). Extending this work, Evans and Fisher (2011) found that metacognitive monitoring and control processes allow individuals to maintain the accuracy of their memory reports over time. They tested participants’ memory for a crime event immediately, and after a 1-week delay. Participants were more likely to provide coarse-grain responses to questions, or refrain from responding altogether after the delay. Such responding decreased the level of detail participants provided, but helped maintain the accuracy of their reports.

Metacognitive monitoring and control processes demonstrably aid individuals in balancing the competing demands for informative, but accurate memory reports. However, the efficacy of these processes has only been examined in relation to recall that occurs in experimental settings free of potential social influence. Yet, remembering often occurs in the presence of others, and research demonstrates that various forms of social influence can affect memory performance. For example, Betz et al. (1996) asked participants to read a story and complete a recognition task. During the recognition task, participants were exposed to bogus tallies representing how many of six other participants selected each of the response options. On a subsequent cued recall task, participants were more likely to provide answers selected by the implied majority, especially for less-memorable, non-distinctive items. Underscoring the persuasiveness of this information source, the effect persisted even when participants were instructed to ignore the answers provided by others. These findings demonstrate one type of social influence effect—that of conformity—on memory.

Bless et al. (2001) explored the boundary conditions of social comparison effects on memory processes and found that low confidence in one’s own memory appears to increase the tendency to engage in social comparison. When participants were not confident that their lack of recall for a stimulus indicated its absence from a previously studied list, they tended to rely on others to determine whether or not the stimulus had indeed been presented. This susceptibility to social influence was dependent on conditions such as exposure time and the salience of stimuli. Sub-optimal encoding of details therefore seems to increase the influence of social factors on subsequent recall. Even perceptions of encoding quality can increase rememberers’ susceptibility to social influence (Gabbert et al., 2007). Gabbert et al. (2007) told participants that they had viewed a set of pictures either for half as long, or twice as long as a co-witness. In actuality, participants had viewed a slightly different set of pictures than their co-witness, but for the same amount of time. The participant and the co-witness then discussed the pictures before providing a free recall report. Participants who believed they had viewed the material for a shorter duration were more likely to incorporate incorrect information mentioned by the co-witness into their own accounts.

Gabbert et al.’s (2007) findings extend those of previous studies of social influence factors affecting recall and memory reporting in the eyewitness memory literature. Co-witnesses to a crime frequently discuss the event with each other, and this has been found to influence their subsequent reports (Paterson and Kemp, 2006; Skagerberg and Wright, 2008; Wright et al., 2009). Social information provided by authorities in the form of feedback can also affect witnesses’ confidence in their recall, and their judgments regarding the quality of the witnessing experience (goodness of view, duration of encoding time, etc.; Wells and Bradfield, 1998; Dixon and Memon, 2005; Leippe et al., 2006). The present research is concerned specifically with social feedback effects on recall (but for a meta-analysis of feedback effects on recognition see Douglass and Steblay, 2006).

The existing literature provides some guidance regarding the effects of feedback on the accuracy of individuals’ memory reports, and their reported confidence. Roper and Shewan (2002) tested participants’ recall before and after labeling them as ‘good’ or ‘poor’ eyewitnesses. These labels were randomly assigned, and did not reflect participants’ genuine performance. Providing participants with positive feedback (‘good’ label) improved their recall performance on a second assessment. Participants who received negative feedback (‘poor label’) were more likely to comply with leading questions. In another study, participants viewed a video of a staged robbery and made a forced-choice identification of the perpetrator from a target-absent line-up (i.e., all identifications were incorrect). The experimenters then informed half of the participants that they were ‘good eyewitnesses,’ who had correctly identified the perpetrator, and the other half of participants that they were ‘poor eyewitnesses,’ who had made an incorrect identification (Dixon and Memon, 2005). After receiving this feedback verbally and in writing, participants were asked to provide details of the crime and perpetrator. Participants who received negative feedback expressed decreased confidence in the accuracy of their recall, yet this decrease in confidence did not affect the quantity or accuracy of information provided. The authors concluded that feedback concerning recall exerts an effect on eyewitness’ confidence in their memory.

While the feedback provided in Roper and Shewan (2002) and Dixon and Memon (2005) was self-relevant (in that participants were given a direct evaluation of their own performance) and categorical, feedback of a comparative nature can also affect individuals’ confidence in their memory and their subsequent memory reports. Leippe et al. (2006) gave participants either negative or positive feedback regarding the accuracy of their memory reports – relative to a co-witness – for a videotaped theft. Participants who received positive comparative feedback later made faster identifications with increased accuracy, and reported a higher level of confidence in the accuracy of their recall than participants who had received no feedback. Despite being associated with decreased confidence, negative feedback did not slow down or render participants’ reports less accurate. The authors concluded that participants’ belief in the accuracy of their memory was affected by the social comparative feedback. Positive feedback boosted belief in memory accuracy, while negative feedback lowered it, as measured by confidence. Consistent with other work, these changes in memory confidence were reflected in participants’ retrospective reports of the witnessing experience (Wells and Bradfield, 1998; Douglass and Steblay, 2006).

In sum, the results of research on how social feedback affects eyewitness recall indicate that positive feedback can increase individuals’ confidence in the accuracy of their reports, while negative feedback can decrease confidence (Roper and Shewan, 2002; Dixon and Memon, 2005; Leippe et al., 2006). Confidence assessments underlie metacognitive decision-making regarding which details of a memory rememberers choose to report (Koriat and Goldsmith, 1996). However, aside from effects on confidence, research has yet to examine the effects of social comparative feedback on metacognitive mechanisms underlying the selection of information for reporting. The aim of the present research was to investigate whether, in addition to accuracy and quantity of information volunteered, social comparative feedback could also affect the precision with which individuals choose to report details from memory. Understanding how extraneous factors such as social comparative information gleaned from a co-witness or investigative interviewer affect the metacognitive processes underlying memory reporting could lead to more theoretically informed interviewing approaches, and a better appreciation of eyewitness memory performance.

In two experiments, we examined the effect of receiving social comparative feedback regarding a co-witness’ or one’s own memory performance on participants’ subsequent memory reports. We introduced a social manipulation (the provision of social comparative feedback) with the expectation that it would affect metacognitive monitoring and control processes, and participants’ resulting memory reports. Experiment 1 investigated the influence of participants’ perception of the accuracy and informativeness of a co-witness’ memory on the confidence and level of detail they reported regarding a witnessed event. Participants received either positive or negative feedback about the quality of a co-witness’ report, before being asked to answer questions about a videotaped crime. In Experiment 2, participants watched a crime video and completed a “practice task” that involved answering a set of questions about a character from the video. Participants were then given self-relevant feedback pertaining to their performance on the practice task, before answering further questions about the crime. We expected that giving participants negative or positive feedback about their own memory performance would affect their confidence in the accuracy of their memory, and therefore also affect the level of detail, or grain size, of the information they chose to report. In line with findings from other studies of social comparative feedback effects on memory reporting, we did not expect the accuracy of participants’ reports to be affected in either of the two experiments (Dixon and Memon, 2005; Leippe et al., 2006). The present experiments extend the existing literature on both social influences and metacognitive processes affecting memory by examining the two phenomena jointly.

## Experiment 1

### Method

#### Design

In a between-subjects design, participants were randomly allocated into one of three conditions: high co-witness score feedback, low co-witness score feedback, and control (no score feedback). We manipulated exposure to the score of a co-witness and examined the effect of that exposure on participants’ (a) confidence in the accuracy of their recall for a crime video, (b) actual recall accuracy, (c) volunteering of fine-grain and coarse-grain details, and (d) withholding of responses to cued recall questions.

#### Participants

Participants (*N* = 87) were university students or employees (65 females; Age *M* = 27.5, *SD* = 12.4) who were recruited through the department of Psychology’s research pool and a database of individuals who signed up to receive information about research participation. Conditions for participation included having normal or corrected-to-normal vision (as assessed by self-report), and being over the age of 18 years. Participants were given course credit for participating or were paid a small honorarium. Ethical approval for the experiment was obtained from the University’s Science Faculty Research Ethics Committee.

#### Materials

##### Stimulus Event

The stimulus event was a 1-min video depicting a (simulated) burglary. In the video, two young men cycle up to a house and forcibly enter through a back door. Once inside, the perpetrators steal a laptop and some money before making their escape (Tehguns, 2011). Participants in the control condition viewed the video individually, while those in the experimental condition viewed the video in pairs. After viewing the video on a computer screen, all participants completed a written filler task (approximately 5 min), after which they completed the recall task.

##### Recall Task

The recall task consisted of 23 cued recall questions. The questions related to details from the video (e.g., How old was the perpetrator who broke into the house? What color was his top? What color was the laptop the perpetrators stole? How many drawers did they open?). Following Koriat and Goldsmith (1996) and Weber and Brewer (2008), questions were presented in two phases. In Phase I, participants provided a coarse- and a fine-grain response to each question (forced report). For the purpose of easily eliciting these types of answers, questions required either numeric answers or referred to the color of an object in the video, as in Weber and Brewer (2008). No specific guidance was given regarding how ‘coarse’ numeric responses could be, participants were simply asked to provide a range (e.g., 17–20 years old). Fine-grain responses to questions with numeric answers were restricted to specific whole numbers (e.g., 27 years old). Coarse-grain responses to questions about the color of objects were restricted to shades (dark, light, warm, and cool). Finally, fine-grain responses to questions about the color of an object were restricted to a specific color (e.g., red, white). Participants were also asked to rate their level of confidence in the accuracy of each of the fine- and coarse-grain answers they provided on a scale of 0–100% (increasing confidence) in increments of 10%. The order of the questions was randomized, and the order in which participants were asked to enter fine-grain and coarse-grain answers was counterbalanced.

In Phase II, participants were presented with the same questions, along with the coarse- and fine-grain answers they provided in Phase I (without their original confidence ratings) as response alternatives. They were instructed to imagine that they were making a statement to the police with regard to the witnessed crime, and to select the response alternative (fine-grain or coarse-grain) that they would give to investigators. Participants were also explicitly told that they could withhold responses (e.g., by answering ‘don’t know’) if they were unsure of the correct answer. They were told to be as accurate as possible without guessing.

#### Procedure

Prior to the start of Phase 1, participants in the experimental groups saw either ‘high’ or ‘low’ feedback about the co-witness’ performance. This bogus feedback was presented in the form of a test percentile that was prominently displayed in the center of the computer screen, and supposedly referred to overall accuracy of details volunteered by the co-witness at Phase II. Participants in the high score feedback group saw a high accuracy co-witness score (i.e., 93%). Conversely, participants in the low score feedback group saw a low accuracy co-witness score (i.e., 28%). We exposed participants to the score of an implied co-witness to give them the impression that the co-witness had performed either very well (high score feedback condition) or poorly (low score feedback condition). In fact, the experimenter fabricated all scores. The manipulation was incidental in nature; that is, participants were not overtly instructed to take notice of the score. Instead, after signing the informed consent, viewing the video, and completing the filler task in separate rooms, participants were led into another room by the experimenter. The purpose of moving participants into another room was to give them the impression that the co-witness had completed the recall task in the second room while they had been working on the filler task in the first room. After giving the participants about 5–10 s to look at the computer screen, the experimenter instructed the participant to click the ‘next’ button to begin the recall task. We deliberately did not draw the attention of participants in the co-witness condition to the apparent score of their co-witness. Our aim was to replicate a situation in which social feedback might be obtained indirectly in a natural way (rather than more explicitly instructed for – which is a feature of previous research). We also wished to avoid a situation in which our social feedback manipulation might have been transparent to participants – this would almost inevitably have been the case with a direct instruction.

Participants in the experimental groups were asked if they had noticed the co-witness’ score at the start of the experimental session in a manipulation check at the end of the recall task. Participants were also asked what they thought the purpose of the experiment was. The complete procedure took approximately 30 min. On completion, all participants were thanked. Participants were debriefed once data collection was completed.

#### Coding

The principal investigator (PI) and an independent rater determined what constituted accurate answers to the 23 items in the recall task. This was done by watching the video, answering the questions individually, and then comparing results. Disagreements were discussed, and more than one correct answer accepted where individual answers could not be reconciled (e.g., both blue and black for the color of the perpetrators trainers). A fine-grain answer was considered correct if it matched the answer to the question that was agreed upon by the investigator and an independent rater. A coarse-grain answer was considered correct if it contained the agreed upon answer (e.g., correct answer of three items stolen from the house is contained in the coarse-grain answer range of “2–5”). After accurate answers were determined, the PI and rater separately coded the data for accuracy in a spreadsheet with condition identifiers removed for all participants. Inter-coder agreement was high, with a single intraclass correlation (ICC) value of 0.90.

### Results

Two participants were removed because their scores were outliers (more than three standard deviations away from the mean) for two or more of the dependent variables. A third participant’s information was excluded due to failure to follow instructions for completing the recall task. Data from the remaining 84 (control = 30; high score feedback = 27; low score feedback = 27) participants was entered into the first analysis.

In line with previous research, preliminary analyses revealed a positive correlation between confidence in fine-grain answers at Phase I, and volunteering of fine-grain answers at Phase II, *r*(82) = 0.49, *p* < 0.01. A one-way analysis of variance (ANOVA) revealed that there was no effect of condition (receiving high, low, or no social comparative feedback) on participants’ (a) confidence in the accuracy of their fine-grain answers at Phase I, *F*(2, 83) = 0.57, *p* = 0.57, ω = 0.10; (b) accuracy of both fine and coarse-grain responses at Phase I, *F*(2, 83) = 0.30, *p* = 0.74, ω = 0.14; (c) volunteering of fine-grain responses, *F*(2, 83) = 2.12, *p* = 0.13, ω = 0.16 and (d) withholding responses at Phase II, *F*(2, 83) = 1.12, *p* = 0.33, ω = 0.05.

A manipulation check revealed that 15 of the 54 participants in the two experimental groups did not notice the manipulation (co-witness score). These 15 cases were excluded from the second analysis, which left a total of 69 participants (control = 30; high score feedback = 19; low score feedback = 20). To check whether participants in the experimental group who had not noticed the manipulation were biasing the results, we examined group means, standard deviations and confidence intervals for all dependent variables after these cases were removed (see **Table 1**).

**Table 1:** Experiment 1: Descriptive statistics for dependent variables by condition after removal of data from experimental participants who did not notice the manipulation.

	Control (*n* = 30)		Low score feedback (*n* = 19)		High score feedback (*n* = 20)	
	*M (SD)*	95% CI	*M (SD)*	95% CI	*M (SD)*	95% CI
Fine-grain confidence^a^	62.7 (13.4)	[57.7; 67.7]	66.4 (9.7)	[61.9; 71.0]	67.7 (8.0)	[63.9; 71.5]
Fine-grain volunteering^b^	0.37 (0.13)	[0.33; 0.42]	0.46 (0.13)	[0.39; 0.51]	0.44 (0.14)	[0.37; 0.51]
Responses withheld^c^	0.20 (0.14)	[0.14; 0.25]	0.15 (0.11)	[0.10; 0.20]	0.15 (0.12)	[0.09; 0.21]
Overall accuracy^d^	0.68 (0.12)	[0.64; 0.73]	0.68 (0.10)	[0.64; 0.72]	0.71 (0.10)	[0.66; 0.76]


*^a^Confidence in fine-grain answers at Phase I (0–100%). ^b^Proportion of fine-grain answers volunteered at Phase II. ^c^Proportion of responses withheld at Phase II. ^d^Proportion of accurate of fine and coarse-grain responses volunteered at Phase I*.

High and Low score feedback group means were very similar for all dependent variables, but differed from means for the control group. To test whether this difference was statistically significant, we collapsed data from the high and low score feedback groups and ran independent samples *t*-tests. Feedback group (experimental *n* = 39; control *n* = 30) was entered as the independent variable, with confidence, accuracy, withholding of responses and fine-grain volunteering as dependent variables. Results revealed a main effect of experimental condition on the volunteering of fine-grain responses at Phase II. On average, participants who had viewed a co-witness’ score prior to starting the recall task volunteered a higher proportion of fine-grain answers at Phase II (*M* = 0.45, *SD* = 0.13) than participants in the control condition (*M* = 0.37, *SD* = 0.13). This difference was significant, *t*(67) = -2.33, *p* = 0.023 and represented a medium sized effect, *d* = 0.57. There were no significant differences between group means for any of the other dependent variables (see **Table 2**). The significant positive correlation between confidence in fine-grain answers at Phase I and volunteering of fine-grain answers at Phase II was slightly reduced in this smaller sample, *r*(67) = 0.40, *p* < 0.01.

**Table 2:** Experiment 1: Descriptive statistics for dependent variables by condition after collapsing data from participants in the high and low score feedback experimental groups.

	Control (*n* = 30)		Experimental (*n* = 39)	
	*M (SD)*	95% CI	*M (SD)*	95% CI
Fine-grain confidence^a^	62.7 (13.4)	[57.7; 67.7]	67.0 (8.8)	[64.2; 70.0]
Fine-grain volunteering^b^	0.37 (0.13)	[0.33; 0.42]	0.45 (0.13)	[0.40; 0.49]
Responses withheld^c^	0.20 (0.14)	[0.14; 0.25]	0.15 (0.12)	[0.11; 0.18]
Phase I CG accuracy^d^	0.73 (0.12)	[0.68; 0.77]	0.75 (0.11)	[0.72; 0.79]
Phase I FG accuracy^e^	0.64 (0.15)	[0.59; 0.70]	0.64 (0.10)	[0.60; 0.67]
Overall accuracy^f^	0.68 (0.12)	[0.64; 0.73]	0.70 (0.10)	[0.66; 0.73]


*^a^Confidence in fine-grain answers at Phase I (0–100%). ^b^Proportion of fine-grain answers volunteered at Phase II. ^c^Proportion of responses withheld at Phase II. ^d^Proportion of accurate coarse-grain responses volunteered at Phase I. ^e^Proportion of accurate fine-grain responses volunteered at Phase I. ^f^Proportion of accurate fine- and coarse-grain responses volunteered at Phase I*.

### Discussion

Experiment 1 examined the effects of receiving social comparative feedback about the quality of a co-witness’ recall for a jointly encoded event on participants’ metacognitive monitoring and control strategies in a subsequent memory report. Participants’ confidence in the fine-grain (detailed) responses they provided at Phase I, as well as their likelihood of volunteering these responses at Phase II, were examined in relation to the type of feedback given. We expected that giving participants negative or positive feedback about a co-witness’ memory performance would influence their confidence in the accuracy of their own memory. While descriptive statistics did reveal higher mean fine-grain confidence ratings for the experimental groups (high and low score feedback) than the control group (no score), this group difference was not significant. It is possible that feedback may not have significantly affected confidence because it was not self-relevant—unlike the feedback participants were provided in Dixon and Memon (2005), Leippe et al. (2006), which was. Despite there being no significant difference between group with respect to confidence, participants who saw either a high or low co-witness score (cf. viewing no feedback information) before completing the recall task volunteered significantly more detailed (fine-grain) responses Phase II. In line with findings from the literature on metacognitive regulation and reporting in memory, participants’ volunteering of fine-grain answers at Phase II was positively correlated with confidence in the accuracy of these answers at Phase I across conditions (see Goldsmith et al., 2002; Weber and Brewer, 2008).

An awareness of assessment and potential for comparison may have increased participants’ motivation to provide detailed answers. If participants saw the co-witness score and anticipated that the accuracy of their own performance would be scored, one might expect that they would select more coarse-grain responses at Phase II. An emphasis on accuracy would be better served by an increase in coarse-grain responses, which are of a wider range margin and are therefore more likely to be accurate. However, it may be that participants relate accuracy to precision, and thus felt that selecting fine-grain responses at Phase II would improve the overall quality of their report. It is also possible that introducing a social element activated communication norms, which increased participants’ emphasis on informativeness (Grice, 1975; Yaniv and Foster, 1995, 1997; Blank, 2009). As Ackerman and Goldsmith (2008) observed, individuals strive to achieve criterion levels of informativeness, at times violating their criterion for accuracy in order to do so. Other researchers have found that participants avoid giving coarse-grain responses, and that this may be particularly true in social exchanges, where these responses are perceived as violating implicit norms of communication (Yaniv and Foster, 1995, 1997; McCallum et al., 2016).

The presence of another individual in the experimental conditions may also explain the observed effect. According to the drive theory of social facilitation (Zajonc and Sales, 1966), the mere presence of others during task performance can increase arousal, which leads to an increase in the frequency of the dominant response in a given context. Goldsmith et al. (2002) propose that the fine-grain answer is the default response, due to its perceived informativeness. The presence of a co-witness may therefore be responsible for the observed increase in participants’ volunteering of fine-grain responses.

Participants may also have been more likely to risk providing detailed, but potentially incorrect answers due to the lack of immediate performance-related consequences in a voluntary, lab-based study. Unlike recall tasks undertaken in a laboratory setting, the accuracy and precision of information reported in a real life investigative interview is of great consequence. There were no drawbacks to volunteering more detailed but potentially inaccurate information in the experiments presented here. In real life, reporting detailed information that is likely to be inaccurate can negatively impact a criminal investigation. Furthermore, Yaniv and Foster (1997) proposed that metacognitive decision making during memory reporting may be influenced by differences in timing of payoffs for informativeness and accuracy. Precision and informativeness are often immediately rewarded, whilst overly coarse answers are not. The accuracy/inaccuracy of answers becomes evident at a later time, and therefore participants are encouraged to report a grain-size that increases immediate gain. In this case, that gain might relate to a perceived increase in informativeness—which individuals strive for—through the provision of fine-grain details (Ackerman and Goldsmith, 2008). To our knowledge, this is the first study to assess the effect of social comparative feedback on the grain size of participants’ responses. We therefore cannot compare the increase in volunteering of fine-grain details observed here with the results of other feedback studies we have reviewed. Future studies should examine the effect of immediate and delayed consequences/rewards for responding on the precision of participants’ memory reports.

Consistent with previous work, the accuracy of participants’ memory reports in Experiment 1 was unaffected by the feedback manipulation (e.g., Dixon and Memon, 2005; Leippe et al., 2006). However, the findings from Experiment 1 do not replicate the effects of feedback on confidence found in previous studies (Dixon and Memon, 2005; Leippe et al., 2006). It is possible that this is because the feedback provided was not directly self-relevant, but pertained to the performance of the co-witness.

The increase in fine-grain responding by participants in the experimental groups may have been motivated by a desire to outperform the co-witness, or even the expectation of receiving self-relevant social comparative feedback after completing the recall task. The small effect size for the main finding may have been due to the subtlety of the incidental manipulation; 14 participants in the experimental condition reported that they did not notice it. Therefore, in Experiment 2, we sought to increase the saliency of the social feedback manipulation. Additionally, to control for the possibility that expectation of feedback was leading to the observed increase in fine-grain responding, participants in Experiment 2 were informed (via onscreen instructions) that their performance on the cued recall task would not be scored.

## Experiment 2

Experiment 2 investigated the potential effects of receiving salient, self-relevant, social comparative feedback following a practice recall task on metacognitive regulation and reporting for a subsequent recall task. In this experiment, we sought to isolate any potential effects of social comparison on metamemory from the effects of expecting performance feedback by deliberately informing participants that their performance on the cued recall task that followed the practice task would not be scored.

After viewing a video of a mock crime event, participants completed a practice task comprised of questions pertaining to one of the characters from the video. After the practice task, participants were given bogus feedback in the form of a percentile score comparing their performance to that of others who had completed the task in terms of both accuracy and level of detail. Thus, unlike Experiment 1, feedback in Experiment 2 was both direct (not incidental in nature) and self-relevant (pertained to the participant’s own performance on a memory task). Providing self-relevant feedback permits a more direct comparison of results from Experiment 2 to those of other studies exploring feedback effects on eyewitness memory reports (e.g., Dixon and Memon, 2005; Leippe et al., 2006). Additionally, to test whether the results of Experiment 1 were due to participants’ expectation that their performance on the cued recall task would be scored, we indicated in the instructions for Experiment 2 that responses on the cued recall task would not be scored. As such, we eliminated expectation of evaluation as a potential confound. We were interested in whether or not telling participants how their memory performance on the practice task compared to that of others would influence their subsequent metacognitive regulation strategy. The format of the cued recall task in Experiment 2 was identical to that used in Experiment 1.

Based on findings from the literature on providing self-relevant social comparative feedback to eyewitnesses (Roper and Shewan, 2002; Dixon and Memon, 2005; Leippe et al., 2006), we predicted that receiving negative feedback would lead to a decrease in participants’ reported confidence in the accuracy of their answers in Phase I, and a corresponding decrease in the volunteering of fine-grain answers in Phase II of the recall task. We also expected that receiving negative feedback would lead to an increase in response withholding (a greater number of “I don’t know” responses) at Phase II. We expected that receiving positive feedback would have the opposite effect, resulting in increased confidence and volunteering of fine-grain details, and decreased withholding of responses. We did not expect the manipulation to have an effect on the accuracy of participants’ memory reports.

### Method

#### Design

In a between-subjects design, we manipulated feedback and examined its effect on reported confidence (0–100%), selection of grain size (coarse vs. fine), response withholding (number of ‘don’t know’ responses), and accuracy in a subsequent memory assessment. Participants were randomly allocated to one of three conditions; High score feedback, Low score feedback, or No feedback/Control.

#### Participants

Ninety undergraduate students participated in this experiment. The sample was comprised of 71 females and 19 males, between the ages of 18 and 39 (*M*_age_ = 22.3; *SD* = 3.4). Participants were recruited through the departmental research participation pool and through advertising flyers posted in various university buildings. They were either paid a small honorarium, or granted course credit. Inclusion and exclusion criteria were the same as in Experiment 1.

#### Materials

##### Stimulus Event

Participants viewed a 3-min video depicting a (simulated) distraction theft. In the video, a man enters the home of an elderly couple claiming to be a government employee who has been sent to check their electricity meter. While he distracts them, an accomplice enters the house and steals a few items from the upstairs bedroom before leaving. The first perpetrator then steals some money from the couple and leaves. In the final scene he is shown getting into a car and driving off (westmerciapolicetv, 2011).

#### Procedure

After viewing the video, participants completed a computerized “practice task.” This practice task was comprised of six questions about the male victim in the video (e.g., How old is the male victim?; What color is his shirt?). Participants provided coarse- and fine-grain answers to each question, along with a rating of their confidence in the accuracy of their answers on a scale ranging from 0 to 100% (in 10% increments). At the end of the practice task, experimental participants saw a screen with the word “calculating…” displayed just beneath a download status bar that quickly moved from empty to full. Once the download bar was full, the screen displayed either a high (93%) or low (37%) accuracy percentile rank supposedly reflecting the performance of the participant on the practice task. Control participants were not shown a download bar screen or provided with feedback. Thus, in contrast to Experiment 1, in which participants were exposed to feedback supposedly related to the recall performance of a co-participant, in Experiment 2 participants received self-relevant feedback about their own performance on a practice task. This feedback was in fact false, and suggested to participants that they had either performed very well (high score feedback of 93% accuracy) or poorly (low score feedback of 37% accuracy).

After the practice task, participants answered a further 22 questions about the video. They were informed that their performance on the task would not be scored. The structure of the phases in the cued recall task was the same as in Experiment 1. In the manipulation check at the end of the task, participants were asked what they thought the purpose of the experiment was. Participants in the experimental groups were also asked if they believed the score they were shown after the practice task was representative of their performance, and if they thought it had influenced their subsequent recall in any way. The entire procedure took approximately 30 min. Afterward, participants were thanked, and informed that they would receive a debrief email about the purpose of the study once data collection was complete.

### Results

#### Manipulation Check and Data Screening

After screening the data, one case was removed due to a technological error that resulted in most of the participant’s responses not being recorded. Two additional cases were identified as outliers (more than three standard deviations away from the mean on one or more of the dependent variables) and removed. Data from a total of 87 participants remained (34 in the high score feedback group, 30 in the low score feedback group and 23 in the control group).

#### Effect of Feedback on Confidence, Response Volunteering, Response Precision, and Accuracy

We conducted a one-way ANOVA with type of feedback (control, high, or low score feedback) as the independent variable and participants’ confidence in the accuracy of their fine-grain answers at Phase I, as well as the total number of fine-grain responses volunteered and number of responses withheld at Phase II as dependent variables. We found no significant group differences for participants’ (a) confidence in the accuracy of their fine-grain answers at Phase I, *F*(2, 86) = 0.71, *p* = 0.50, ω = 0.08, (b) fine-grain volunteering, *F*(2, 86) = 1.51, *p* = 0.25, ω = 0.11 and (c) responses withholding at Phase II, *F*(2, 86) = 0.59, *p* = 0.57, ω = 0.10.

In one of the items in the manipulation check, we asked participants what they thought the purpose of the study was. A total of 14 participants accurately guessed that the feedback they received was part of the experimental manipulation and/or expressed some suspicion as to its authenticity. The data for these participants was removed for a second analysis. Data from the remaining 73 participants (23 in the control, 26 in the low score, and 24 in the high score feedback group) was entered into a second ANOVA with the same independent and dependent variables. Again, results revealed no significant group differences for participants’ (a) confidence in the accuracy of their fine-grain answers at Phase I, *F*(2, 72) = 0.27, *p* = 0.77, ω = 0.14, (b) volunteering of fine-grain responses, *F*(2, 72) = 0.73, *p* = 0.48, ω = 0.09, and (c) withholding of responses at Phase II, *F*(2, 72) = 0.34, *p* = 0.71, ω = 0.14. **Table 3** displays group means, standard deviations and confidence intervals for all dependent variables entered into this analysis.

**Table 3:** Experiment 2: Descriptive statistics for dependent variables by condition after removal of data from experimental participants who guessed the manipulation.

	Control (*n* = 23)		Low score feedback (*n* = 26)		High score feedback (*n* = 24)	
	*M (SD)*	95% CI	*M (SD)*	95% CI	*M (SD)*	95% CI
Fine-grain confidence^a^	62.8 (13.3)	[57.1; 68.6]	65.2 (10.5)	[61.0; 69.5]	63.6 (12.5)	[58.3; 68.8]
Fine-grain volunteering^b^	0.40 (0.11)	[0.35; 0.44]	0.43 (0.11)	[0.38; 0.47]	0.39 (0.14)	[0.33; 0.45]
Responses withheld^c^	0.20 (0.13)	[0.15; 0.26]	0.17 (0.16)	[0.11; 0.24]	0.20 (0.14)	[0.15; 0.26]
Overall accuracy^d^	0.65 (0.10)	[0.61; 0.70]	0.68 (0.11)	[0.63; 0.72]	0.68 (0.09)	[0.64; 0.71]


*^a^Confidence in fine-grain answers at Phase I (0–100%). ^b^Proportion of fine-grain answers volunteered at Phase II. ^c^Proportion of responses withheld at Phase II. ^d^Proportion of accurate fine and coarse-grain responses volunteered at Phase I*.

### Discussion

The results of Experiment 2 are contrary to our hypotheses. Participants in the control, high, and low score feedback groups did not differ on any of our key measures. There are several potential explanations for the lack of an effect of feedback on responding.

The manipulation check questions indicated that while most experimental participants were accepting of negative feedback, many were suspicious of positive feedback. This is not altogether surprising, as research has shown that some people exhibit a stable tendency to distrust their memory, or *trait memory distrust* (Van Bergen et al., 2009). One study estimated that at least 10% of the population has a tendency toward pessimistic evaluations of their memory capacity in comparison to that of others (Crombag et al., 2000). All participants who expressed suspicion about the authenticity of the score in the manipulation check were eliminated from the second analysis. However, it is possible that even those participants who did not express suspicion/guess the manipulation as reported in the manipulation check may not have been entirely accepting of the feedback score they received, which may have weakened the effect of the experimental manipulation.

Another possibility is that participants’ performance on the second set of questions was unaffected by feedback because they were told that the second set of questions would not be scored. This was a deliberate methodological decision made during the design of the study to rule out the possibility that expectations about evaluations would lead to an increase in fine-grain responding. We wanted to isolate the potential effects of the self-relevant social comparative feedback provided for the practice task on subsequent metacognitive regulation of memory reporting. According to Feedback Intervention Theory, when individuals receive negative feedback, they are likely to increase their efforts to improve if given the opportunity on a subsequent task (Kluger and DeNisi, 1996). If the feedback received is positive, with room for improvement, performance efforts may also increase (Kluger and DeNisi, 1996). Thus, in the present experiment, (a) participants were not given a second opportunity to assess their performance and (b) those who received high score feedback were not left with much room for improvement. In hindsight, the feedback manipulation may have been ineffectual for these reasons. This issue is discussed further in the General Discussion.

Finally, in contrast to Experiment 1, all participants in Experiment 2 viewed the stimulus video individually. In Experiment 2, the presence of co-witnesses was merely implied. If social facilitation underpinned the effect found in Experiment 1, then failure to replicate in Experiment 2 would not be surprising. This highlights the need for research on how the mere presence of others affects meta-memory.

## General Discussion

The present research investigated the effects of receiving social comparative feedback regarding the recall performance of a co-witness and oneself on participants’ subsequent recall. In Experiment 1, receiving feedback of any level (high or low score) regarding the performance of a co-witness on a recall task increased the number of fine-grain (detailed) responses reported by participants on a subsequent memory assessment. In Experiment 2, receiving self-relevant feedback on a practice memory task did not affect participants’ confidence in the accuracy of their recall, or the level of detail they provided in a subsequent memory report. While we expected that receiving self-relevant feedback in Experiment 2 would replicate and increase the effect observed in Experiment 1, this was not the case. Several of the participants who received positive feedback expressed doubts relating to the accuracy of this assessment of their performance. Additionally, participants’ responses on the cued recall task that followed the feedback may have been unaltered because participants were told that their performance would not be scored a second time. Thus, participants may have had no motivation to increase the level of detail they provided following the practice task. In Experiment 1, seeing a co-witness’s score may have led participants to believe that their own performance would be scored, thereby increasing their motivation to provide a detailed memory report. In Experiment 2, we informed participants that their performance on the cued recall task would not be scored because we predicted social influence effects irrespective of evaluation concerns, but this was not the case.

It is interesting to note that in Experiment 1, the experimental groups did not express significantly higher confidence in the accuracy of their fine-grain responses than the control group. However, participants in the experimental groups did volunteer significantly more fine-grain answers than the control group. According to the revised dual-criterion model, fine-grain responses are volunteered when confidence in the accuracy of these responses is high (Ackerman and Goldsmith, 2008). While there was a positive correlation between confidence in the accuracy of fine-grain responses at Phase I and fine-grain volunteering at Phase II, the magnitude of the correlation was medium, suggesting that there were other factors influencing participants’ decision to volunteer fine-grain responses. Possible candidates for further investigation include mere presence effects and increased motivation resulting from expectation of feedback. Another interesting avenue for future research would be to examine the effects of direct (socially) encountered feedback on metacognitive monitoring and control processes. While previous studies have successfully demonstrated feedback and conformity effects via computerized delivery of feedback and implied co-witnesses, effects may be stronger with direct interaction (Betz et al., 1996; Kluger and DeNisi, 1996).

Finally, the sample sizes in both Experiments 1 and 2 were reduced after manipulation checks, which may have limited the power of our statistical analyses. We chose our original sample sizes based on those used in similar studies. In Leippe et al. (2006) the number of participants per condition was approximately 27 (experiment 1) and 21 (experiment 2) participants to a cell. In developing and testing the revised dual criterion model Ackerman and Goldsmith (2008) included 24 participants per experiment. Similarly, Weber and Brewer (2008) included 31 (experiment 1) and 36 (experiment 2) participants. As such, our sample size is in line with previous published research. However, we ran a *post hoc* power analysis using Gpower, and found that a sample of 159 participants is needed to detect an effect size of 0.25 on confidence, with a power of 0.80. This effect size was chosen based on effect sizes (etas) of 0.26 and 0.27 reported in Leippe et al. (2006) for significant ANOVAs assessing the effect of social comparative feedback on participants’ reported confidence in the accuracy of their recall. In light of this finding, we recommend that future research investigating social influences on metamemory include larger sample sizes.

The results of Experiments 1 and 2 do not provide a definitive answer regarding the mechanisms that underlie the observed effects of receiving social comparative information on participants’ subsequent memory reports. However, they do highlight the potential for social comparison to affect the metacognitive appraisals that influence memory output. These studies represent the first attempt to examine the effects of social comparison in this area. Further work must establish the most effective methodologies for investigating the effects of social comparison, and aim to disentangle what are likely to be complex relationships between the effects of evaluation and social comparison. Future studies should also investigate how the mere presence of a co-witness can affect eyewitness’ confidence in the accuracy of their recall, and the amount/degree of detail they choose to report, as well as whether these effects are strengthened through face-to-face interaction.

## Ethics Statement

This research was carried out in accordance with the recommendations of the Concordat to Support Research Integrity and the RCUK Policy and Guidelines on Governance of Good Research Conduct (February 2013). Furthermore, this research complies with the British Psychological Society’s (BPS) Code of Ethics and Conduct (2009) and Code of Human Research Ethics (2014) with written informed consent from all subjects. All subjects gave written informed consent in accordance with the Declaration of Helsinki. The protocol was approved by the Science Faculty Research Ethics Committee of the University of Portsmouth.

## Author Contributions

JR collected the data for these experiments as part of her doctoral thesis work. All listed authors contributed to the design of these experiments to varying degrees, reflected in his/her position on the list of authors. JR wrote most of the manuscript, while JS, LH, MS, and JO made various edits, comments, and recommendations. JR and JS contributed to the analysis of the data. HM reviewed and gave final comments on the penultimate draft of the manuscript.

## Conflict of Interest Statement

The authors declare that the research was conducted in the absence of any commercial or financial relationships that could be construed as a potential conflict of interest.
